# Case of Transposition of the Heart, Stomach, Liver, Amd Spleen—with Rupture of the Heart

**Published:** 1844-11-02

**Authors:** Samual W. Logan

**Affiliations:** The Parish St. Charles, La.


### CASE OF TRANSPOSITION OF THE HEART, STOMACH, LIVER, AMD SPLEEN—WITH RUPTURE OF THE HEART.

By SAMUAL W. LOGAN, M. D. of the Parish St. Charles, La.

The negro Alexander, of strong muscular developement, aged about 26 years, the property of Mr. E. F., of this parish, had frequently come under my care, for the last nine or ten years with diseases incident to the climate, especially those of the digestive organs.—His appetite was always good, and even voracious; and he was accused by several of dirt-eating, which was rendered extremely probable, by the dark gritty matter which he sometimes voided by stool. On every occasion, however, on which he was presented to me, I was invariably struck by a remarkable pulsation in the right side of the thorax, with a total absence of it on the left. For the last two years of his life, he seemed to enjoy good health, and I lost sight of him, until the 27th of October last, when he came again under my care, but with an attack of intermittent fever, which was very prevalent at that time. The paroxysm presented nothing remarkable, and I left him, recommending the administration of the sulphate of quinine, as soon as the attack was fairly over. At an early hour the next morning, I received a note from his master, stating that the negro had died suddenly in the course of the night, on the termination of the paroxysm. I forthwith proceeded to the plantation, in order to examine the body, which I did in presence of M. Edmond Fortier, senior—his son, M. Septime Fortier, and M. Aimé. The following were the appearances which presented themselves twelve hours after death.

On opening the abdomen, I was struck with the enormous developement of the *liver* which occupied the entire upper portion of that cavity, but upon inspecting it more carefully, I found to my astonishment that it was placed entirely on the *left side*, with the large lobe extending over to the right. Its colour was natural, and upon cutting into its substance it was found of a healthy appearance. Upon further examination, I discovered the *stomach* situated in the *right hypochondrium*, with its pyloric orifices extending to the left. The spleen was also located on the *right side*, but small, and divided into *four parts*, joined together by cellular membrane. The other viscera of the abdomen were in their natural situation.—The examination of the thorax exhibited the residence of the heart on the *right side!* The subject had come to his death by rupture of the right auricle of this organ. Extensive adhesions of the pericardium and heart had taken place.—*N. O. Med. Journal.*

